# Phase I dose-escalation study of oral vinflunine in combination with erlotinib in pre-treated and unselected EGFR patients with locally advanced or metastatic non-small-cell lung cancer

**DOI:** 10.1007/s00280-013-2342-3

**Published:** 2013-11-13

**Authors:** M. Krzakowski, J. Bennouna, E. Dansin, D. Kowalski, S. Hiret, N. Penel, S. Favrel, J. M. Tourani

**Affiliations:** 1 Institute of Oncology, Warsaw, Poland; 2 Institut de Cancerologie de l’Ouest-site René Gauducheau, Saint Herblain, France; 3 Centre Oscar Lambret, Lille, France; 4 CHU La Miletrie-University of Poitiers, Poitiers, France; 5 IRPF, Toulouse, France

**Keywords:** Erlotinib, Non-small-cell lung cancer, Phase I, Oral vinflunine

## Abstract

**Background:**

Erlotinib, the epidermal growth factor receptor tyrosine kinase inhibitor, and the intra-venous vinflunine vinca alkaloid microtubule inhibitor have been shown to be effective in the setting of non-small-cell lung cancer (NSCLC) palliative patients with acceptable toxicities. This phase I study was conducted to determine the maximal tolerated dose (MTD) and the safety of an all-oral combination. A potential pharmacokinetic drug–drug interaction was also investigated.

**Patients and methods:**

Patients with unresectable stage IIIB or stage IV NSCLC who failed one or two previous chemotherapy regimens were treated with flat doses of oral vinflunine from day 1 to day 5 and from day 8 to day 12 every 3 weeks and erlotinib daily on a continuous basis. The dose levels of vinflunine/erlotinib were 95/100, 115/100, 115/150 and 135/100 mg.

**Results:**

Thirty patients were enroled. The recommended dose was 115/150 mg and the MTD 135/100 mg. Dose-limiting toxicities included grade 3 febrile neutropenia (1 patient) and related death (1 patient). Non-haematologic grade 3/4 toxicities included fatigue, condition aggravated, hypokalaemia, tumour pain, acneiform dermatitis, diarrhoea, hyperbilirubinaemia and pulmonary haemorrhage, in one patient each. Of 25 patients evaluable for tumour response, 2 patients had partial response and 20 patients had stable disease.

**Conclusion:**

The recommended doses for oral vinflunine and erlotinib combination were, respectively, 115 mg/day from day 1 to day 5 and from day 8 to day 12 every 3 weeks and 150 mg/day. There was no mutual impact on pharmacokinetics. The combination was safe but evaluation in phase II is needed to further refine the activity and toxicity that can be expected with prolonged administration of this dose schedule.

## Introduction

Lung cancer is the most frequent neoplasm. Prognosis of patients with lung cancer is poor with relative 1-year survival rates approximately 30 % and 5-year rates around 10 % [1]. Non-small-cell lung cancer (NSCLC) accounts for approximately 80–85 % of all primary lung neoplasm. Surgery is the preferred treatment of patients with early disease but more than 50–60 % of patients present with locally advanced (stage IIIB) or metastatic disease (stage IV) and they are not suitable for surgery. Systemic chemotherapy has demonstrated palliative benefit and modest prolongation of survival, and two-drug platinum-based chemotherapy is now the standard of care for good performance patients with unresectable locally advanced or metastatic disease [2, 3]. Second-line chemotherapy may be considered in patients with progression after initial platinum-based regimen. Single-agent docetaxel and pemetrexed may produce life prolongation and symptomatic improvement compared to best supportive care in some patients, both agents have been approved for use as second-line chemotherapy [4].

Recently, the epidermal growth factor receptor tyrosine kinase inhibitor (EGFR-TKI), erlotinib (Tarceva^®^) was investigated in NSCLC after the failure of first-line or second-line chemotherapy. It prolonged survival, delayed disease progression and decreased symptoms, as compared to placebo in this clinical setting [5]. Vinflunine (VFL) administered intravenously in NSCLC patients previously treated with a platinum-containing regimen showed efficacy similar to docetaxel [6]. In a randomized phase II study, the addition of chronic, intermittent, low-dose vinorelbine to the EGFR inhibitor gefitinib was effective and manageable in NSCLC patients who failed at least two regimens of chemotherapy. The addition of low-dose vinorelbine produced a significantly better 1-year progression-free survival rate (57.1 vs. 21.2 %; *P* = 0.008) [7]. Importantly, VFL is less neurotoxic than vinorelbine [8].

Given that VFL and erlotinib have an acceptable safety profile and have shown efficacy in second-line treatment of patients with advanced NSCLC, we hypothesized that the combination would result in acceptable toxicity and improved efficacy. Furthermore, they are both available as oral drugs. An all-oral combination regimen is an attractive option in the palliative setting of NSCLC patients. The goal of the present study was to determine the doses of oral VFL and erlotinib used safely in combination and to explore the safety and pharmacokinetic profiles of the combination.

## Patients and methods

### Patient selection

Patients with histologically or cytologically confirmed NSCLC unresectable stage III B or stage IV disease or delayed relapse of any stage no longer amenable to surgery or radiotherapy with curative intent were eligible. Other criteria included are as follows: age between 18 and 75 years, Karnofsky performance status ≥70 % and life expectancy ≥3 months. Patients may have had one or two prior lines of chemotherapy regimen and they had to be at least 4 weeks since prior chemotherapy or 6 weeks if the last regimen included bleomycin or mitomycin C. Prior radiotherapy must have been completed at least 4 weeks before study entry. Adequate bone marrow (haemoglobin ≥10 g/dL, absolute neutrophil count (ANC) >1.5 × 10^9^/L, and platelet count >100 × 10^9^/L), renal (serum creatinine < upper limit of normal (ULN) and creatinine clearance ≥50 mL/min) and liver (total bilirubin ≤ ULN, AST and ALT ≤2.5 × ULN, and alkaline phosphatase ≤5 × ULN, except in patients with documented bone or liver metastases) function were required. Patients of childbearing potential had to use a medically acceptable contraception. Patients previously treated for central nervous system metastases were eligible if they were stable by CT scan or MRI without evidence of cerebral oedema and had no requirement for steroids or anticonvulsivants. Patients with pre-existing grade ≥2 peripheral neuropathy or dysphagia according to the National Cancer Institute (NCI) Common Toxicity Criteria (CTC) version 3.0 were not eligible. Patients previously treated with VFL and/or EGFR inhibitor were not eligible. Finally, patients with any underlying medical condition likely to be aggravated by study treatment and patients receiving a concomitant therapy with strong inhibitors or inducers of the cytochrome P450 isoform 3A4 were not eligible. The protocol, patient information and written informed consent were submitted to Independent Ethics Committees according to the participating countries requirement. All patients had to give written personally signed informed consent.

### Criteria for evaluation

Dose-limiting toxicities (DLT) were defined as the occurrence during the first cycle of one of the following: ANC <0.5 × 10^9^/L for ≥7 days, grade 3 or 4 neutropenia concomitant with fever ≥38.5 °C or grade ≥3 infection, platelet count <25 × 10^9^/L or <50 × 10^9^/L with bleeding, grade ≥3 nausea, vomiting or diarrhoea if persistent despite optimal antiemetic or antidiarrhoeal treatment, grade 3 intestinal ileus, any other drug-related grade ≥3 toxicity and any other drug-related adverse event that results in 30 % dosing omission during cycle 1 or a delay ≥2 weeks in the administration of cycle 2.

Maximal tolerated dose (MTD) was defined as the highest dose at which 2 out of 3 or 2 out of 6 patients developed a DLT during the first cycle. The recommended dose (RD) was the dose below the MTD. It was confirmed by accrual of at least 10 new patients up to a total of at least 16 patients at the end of the phase I.

Efficacy was assessed by using RECIST criteria (version 1.0). Tumour assessments were performed at baseline, at the end of cycle 2 and then every 2 cycles.

Safety was assessed by physical examination and laboratory tests twice weekly during cycle 1, then weekly in the following cycle. Toxicity was graded according to the NCI–CTC version 3.0.

### Study design and treatment plan

This was an open-label, European multicentre, dose-escalation phase I study. Primary objective was to determine the MTD of oral VFL administered once a day from day 1 to day 5 and from day 8 to day 12 every 3 weeks in combination with erlotinib given once a day on a continuous basis. Secondary objectives were to assess the safety of the combination, to investigate a potential pharmacokinetic drug–drug interaction and to assess antitumour activity in NSCLC patients. The doses of VFL and erlotinib were escalated in cohorts of 3–6 patients. A cycle was defined as a 3-week period. Each patient had to receive at least one cycle of the combination to be evaluable for MTD determination and two cycles to be evaluable for pharmacokinetics (PK). The dose-escalation scheme is summarized in Table 1.

**Table 1:** Dose-escalation schema

Dose level	Oral vinflunine (mg/day)	Oral erlotinib (mg/day)
1	95	100
2	115	100
3	115	150
4	135	100

Patients received at least 2 cycles of treatment unless unacceptable toxicity, patient’s refusal or disease progression. Patients with response or stable disease after 6 cycles were allowed to continue their treatment at the discretion of the investigator and underwent the same monitoring as during the first 6 cycles.

### Pharmacokinetic

Pharmacokinetics (PK) of VFL and erlotinib involve mechanisms that are common between both agents: absorption through the alimentary tract wall after oral administration and important metabolization by CYP3A4.

In order to evaluate the potential drug–drug interaction, the PK of both drugs had to be assessed alone and in combination. To obtain a PK profile of VFL alone, the first administration of erlotinib in Cycle 1 was delayed on Day 2. Day 1 of Cycle 1 was the VFL.

The effect of erlotinib on the VFL PK was assessed at its steady state. Day 1 of Cycle 2 was the test period.

The reference for the PK of erlotinib was on day 21 when VFL concentrations were at their minimum. Potential effect of VFL on the PK of erlotinib could be expected when concentrations of VFL were at its maximal (D12) and at the steady state of erlotinib (starting on D8). Therefore, PK of erlotinib co-administered with VFL was evaluated on day 12 (Test period).

Pharmacokinetics (PK) of VFL was studied by measuring whole blood concentrations using a fully validated LC–MS/MS method with a lower limit of quantification of 0.25 ng/mL [9]. Erlotinib and its metabolite OSI-420 were quantified in plasma using a fully validated LC–MS/MS method with a LLOQ of 1 ng/mL [10].

Pharmacokinetic parameters (AUC_0−24h_) of VFL, erlotinib and OSI-420 were estimated using non-compartmental calculation methods.

The effect of concomitant administration of one drug on the PK of the other was assessed by building the 90 % CI for the test/reference ratio of geometric means of AUC_0−24h_/dose.

## Results

### Patient baseline characteristics

A total of 30 patients with locally advanced or metastatic NSCLC were enroled in the study between December 2008 and April 2010. Patient baseline characteristics are summarized in Table 2. Twenty-five patients (83.3 %) had received at least one chemotherapy line in the locally advanced/metastatic setting. Out of the 30 patients enroled, 5 patients were pre-treated with vinca alkaloids (vinorelbine), 2 in the neoadjuvant setting and 3 in the advanced disease setting. The majority of the patients had a performance status of 90–100 % (86.7 %).

**Table 2:** Patient baseline characteristics

Number of patients	30
Median age, years (range)	58.1 (47.0–71.1)
Gender M/F	18/12
Performance status	
70	1 (3.3 %)
80	3 (10.0 %)
90	14 (46.7 %)
100	12 (40.0 %)
Histopathological type	
Adenocarcinoma	11 (36.7 %)
Large cell carcinoma	9 (30.0 %)
Other	8 (26.7 %)
Stage at diagnosis	
IIB	2 (6.7 %)
IIIA	3 (10.0 %)
IIIB	10 (33.3 %)
IV	15 (50.0 %)
Extent of disease	
1 organ	12 (40.0 %)
2 organs	12 (40.0 %)
=3 organs	6 (20.0 %)
Disease-free interval (years*)	
<2 years	26 (89.7 %)
=2 years	3 (10.3 %)
Prior chemotherapy	
Neoadjuvant	3 (10.0 %)
Adjuvant	5 (16.7 %)
Advanced	25 (83.3 %)

* Data missing for one patient

### Determination of the MTD and RD

No DLT was reported in the first dose level (95 mg/day VFL + 100 mg/day erlotinib). At the second dose level (115–100), one patient out of the first 3 included experienced a DLT (grade 3 febrile neutropenia). Three additional patients included at this dose level did not experience any DLT. Dose was further escalated to level 3 (115–150), at which no DLT was observed among the first 3 included patients. Therefore, dose escalation was pursued to level 4 (135–100): 2 of the 3 included patients experienced a DLT corresponding to the definition of the MTD (grade 3 febrile neutropenia concomitant with grade 2 asthenia, and death which was considered drug-related). Additional patients were included at dose level 3 (115–150) in order to confirm the RD. Among the 15 newly included patients for a total of 18, 4 patients (22.2 %) experienced a DLT (grade 4 neutropenia and grade 4 diarrhoea; grade 4 pulmonary haemorrhage; grade 4 neutropenia and grade 3 rash acneiform.

### Safety

Haematological toxicity was the most frequent and was per patient: anaemia (96.7 %), neutropenia (36.7 %) and thrombocytopenia (16.7 %). Of note 12 patients (40.0 %) had already grade ≥1 anaemia at baseline. Five patients received blood transfusions during the study. Only 2 patients (6.7 %) experienced febrile neutropenia (at 115–100 and 135–100 mg/day), and one patient experienced neutropenic infection at 115–100 mg/day.

Grade 3 drug-related events per patient included are as follows: anaemia (20.0 %), neutropenia (16.7 %), fatigue (10.0 %), febrile neutropenia (6.7 %), thrombocytopenia, condition aggravated, hypokalaemia, tumour pain and dermatitis acneiform (3.3 %). Grade 4-related events were limited to neutropenia (23.3 %), diarrhoea, hyperbilirubinaemia and pulmonary haemorrhage (3.3 %).

At the RD, among the 18 treated patients, grade 3 events observed were neutropenia in 4 patients, anaemia in 3 patients and thrombocytopenia in 1 patient.

Grade 4 was reported only for neutropenia in 3 patients. Grade 3 drug-related non-haematological events were limited to fatigue, hypokalaemia and dermatitis acneiform in one patient each, and grade 4 events were observed for diarrhoea, hyperbilirubinaemia and pulmonary haemorrhage in one patient each.

Five (16.7 %) patients died during study treatment, and for 2 of them (6.7 %), the relationship to study drug was suspected (disseminated intravascular coagulation and pulmonary haemorrhage).

Drug-related grade 3 or 4 toxicities are summarized according to the dose levels in Table 3.

**Table 3:** Grade 3/4 drug-related toxicities observed in all cycles

Grade ¾ toxicity	Level 1 (*n* = 3)	Level 2 (*n* = 6)	Level 3 (*n* = 18)	Level 4 (*n* = 3)	All (*n* = 30)
Anaemia	–	3 (50 %)	3 (16.7 %)	–	6 (20.0 %)
Neutropenia	1 (33.3 %)	2 (33.3 %)	7 (38.9 %)	2 (66.7 %)	12 (40.0 %)
Thrombocytopenia	–	–	1 (5.6 %)	–	1 (3.3 %)
Febrile neutropenia	–	1 (16.7 %)	–	1 (33.3 %)	2 (6.7 %)
Diarrhoea	–	–	1 (5.6 %)	–	1 (3.3 %)
Condition aggravated	–	1 (16.7 %)	–	–	1 (3.3 %)
Fatigue	–	2 (33.3 %)	1 (5.6 %)	–	3 (10.0 %)
Hyperbilirubinaemia	–	–	1 (5.6 %)	–	1 (3.3 %)
Hypokalaemia	–	–	1 (5.6 %)	–	1 (3.3 %)
Tumour pain	–	–	–	1 (33.3 %)	1 (33.3 %)
Pulmonary haemorrhage	–	–	1 (5.6 %)	–	1 (3.3 %)
Dermatitis acneiform	–	–	1 (5.6 %)	–	1 (3.3 %)

### Efficacy

Partial response was observed and confirmed in two patients (6.7 %), one at dose level 1 (33.3 %) and one (5.6 %) at dose level 3. Stable disease was achieved in 20 patients (80 %) among 25 patients evaluable for tumour response: 2 (66.7 %) at dose level 1, 4 (66.7 %) at dose level 2, 13 (72.2 %) at dose level 3 and 1 (33.3 %) at dose level 4.

### Pharmacokinetic results

For VFL pharmacokinetic assessment, 25 patients and 19 patients were fully evaluable on day 1 of cycle 1 and on day 1 of cycle 2, respectively. Only 15 patients were evaluable on both days. Mean value of AUC_0−24h_ at the RD of 115 mg for VFL/150 mg for ERL was 643 h.ng/mL on day 1 of cycle 1 (*n* = 17, range [266–1,474]) and 720 h.ng/mL on day 1 of cycle 2 (*n* = 12, range [326–1,831]).

**Fig. 1:**
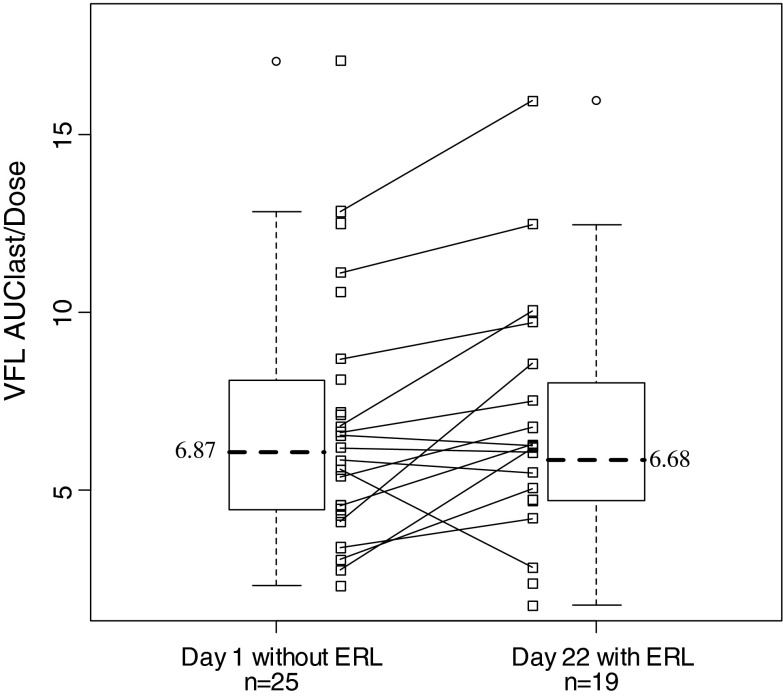
Comparison of VFL AUC_0−24h_ normalized by the dose between days of treatment in all patients included in the study

The test/reference ratio of geometric mean of AUC_0−24h_/dose, calculated on 15 patients (all dose level pooled), was 1.22. The CI 90 % was [1.03–1.43]. When including all available individual PK parameters, the comparison of AUC_0−24h_ normalized by the dose level showed no difference between day 1 and day 22 with similar mean values (Fig. 1).

For erlotinib PK assessment, 23 patients were evaluable on day 12, 18, on day 21 and 18 on both days. Mean value of AUC_0−24h_ at the RD was 3,201 h.ng/mL on day 12 of cycle 1 (*n* = 15, range [649–8,542]) and 2,996 h.ng/mL on day 21 of cycle 1 (*n* = 10, range [987–11,404]).

**Fig. 2:**
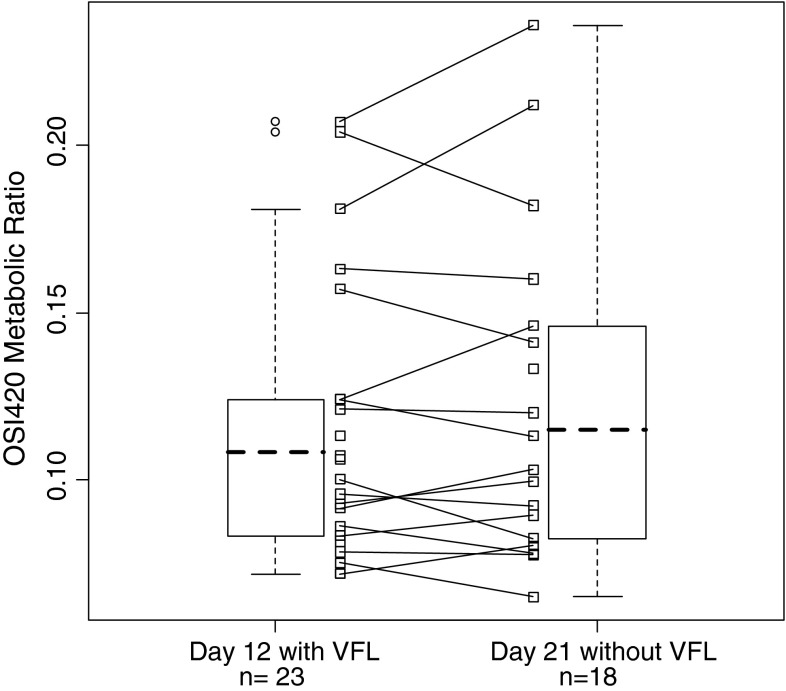
Comparison of OSI-420 metabolic ratio between days of treatment in all patients included in the study

The statistical analysis was performed on 18 patients (all dose level pooled) on AUC/dose of erlotinib. The test/reference ratio was 1.17. The 90 % CI was [1.05–1.31]. The metabolic ratio of OSI-420 formed through CYP3A4 metabolic pathway, was stable between day 12 and day 21 (point estimates of 1.00 with 90 % CI of 0.95–1.05) (Fig. 2).

## Discussion

We report the results of a phase I dose-finding study of VFL orally administered once daily from day 1 to day 5 and from day 8 to day 12 every 3 weeks in combination with erlotinib given orally once daily on a continuous basis in patients with locally advanced or metastatic NSCLC. The RD was defined at 115–150 mg, dose at which 5.6 % of patients achieved a partial response and 72.2 % experienced stable disease.

Oral chemotherapy is considered to be more convenient for patients than intravenous treatment and offers the added advantages of a chronic schedule without the need of intravenous devices. Its potential for enhancing anti-tumour effect and improving patient quality of life is of special interest in the palliative setting of metastatic disease.

However, this combination increases the risk of haematologic toxicity compared to either agent alone. Forty per cent of patients (38.9 % at the RD) experienced grade 3/4 neutropenia. Neutropenia was the main cause limiting delivery of treatment and requiring dose delays. These results are similar to those reported by Davies et al. [11] in a phase I study of erlotinib and vinorelbine in advanced malignant solid tumours and by Scagliotti et al. [12] when combining gefitinib with vinorelbine in a phase II study. Nevertheless, it can be noticed that neutropenia did not translate to a high incidence of severe toxicities, as only 2 patients experienced febrile neutropenia, and one patient experienced neutropenic infection. None of the 18 patients at RD experienced complicated neutropenia.

Both agents are metabolized through cytochrome 3A4, and therefore, potential drug–drug interaction was evaluated in this study. Given higher PK variability with oral drugs and the limited number of patients included into statistical analysis, sufficient power could not be reached to conclude with an acceptance interval of [0.8–1.25]. However, confidence intervals are included in a widened range of [0.7–1.43] commonly used for more variable drugs.

Indeed, the graphical comparison of VFL AUC_0−24h_ normalized by the dose level using data from all patients showed overlapping range of values and no consistent trend of individual values between VFL monotherapy and co-administration with erlotinib.

For erlotinib PK assessment, a drug–drug interaction between both agents would be driven by the inhibition of CYP3A4, and this inhibition would have induced a decrease in the formation of OSI-420 from ERL and as a consequence a decrease in the metabolic ratio of OSI-420 over ERL. However, the confidence interval of this metabolic ratio is fully included in the acceptance range with a test/reference ratio of 1, showing a lack of VFL impact on its formation.

Therefore, a lack of mutual impact on PK when VFL and erlotinib were co-administered can be concluded.

This phase I study shows that oral VFL and erlotinib combination can be delivered safely with acceptable toxicity in unselected patients with locally advanced or metastatic NSCLC. However, more toxicity evaluation in phase II is needed to further refine the real toxicity that can be expected with prolonged administration with this dose schedule.
